# Differences in Psychoneuroendocrine Stress Responses of High-Level Swimmers Depending on Autocratic and Democratic Coaching Style

**DOI:** 10.3390/ijerph16245089

**Published:** 2019-12-13

**Authors:** Manuel Jiménez, Manuel Fernández-Navas, José Ramón Alvero-Cruz, Jerónimo García-Romero, Virginia García-Coll, Iván Rivilla, Vicente Javier Clemente-Suárez

**Affiliations:** 1 Departamento de Didáctica de la Educación Física y Salud, Universidad Internacional de La Rioja, 26002 Logroño, Spain; manuel.jimenez@unir.net (M.J.); Virginia.Garcia@uclm.es (V.G.-C.); ivan.rivilla@unir.net (I.R.); 2 Departamento de Didáctica y Organización Escolar, Universidad de Málaga, 29010 Málaga, Spain; mfernandez1@uma.es; 3 Departamento de Fisiología Humana, Histiología, Anatomía Patológica, y Medicina de la Educación Física y Deportiva, Universidad de Málaga, 29010 Málaga, Spain; alvero@uma.es (J.R.A.-C.); jeronimo@uma.es (J.G.-R.); 4 Faculty of Sports Science, Universidad Europea de Madrid, 28670 Madrid, Spain; 5 Grupo de Investigación en Cultura, Educación y Sociedad, Universidad de la Costa, Barranquilla 080002, Colombia

**Keywords:** coaching styles, cortisol, learning, motivational climate, performance

## Abstract

The aim of the present study was to analyse differences in the psychoneuroendocrine stress responses of high-level, young swimmers depending on whether an autocratic and democratic coaching style was applied. Modifications in cortisol and the motivational climate of 18 young swimmers (15.3 ± 1.86 years, 10 females and 8 males) were analysed before and after two training sessions with equivalent training loads but directed by two coaches applying different approaches, i.e., autocratic (A) and democratic (D). The basal testosterone levels of the coaches were also assessed. The basal testosterone concentration was higher in coach A than in coach D; the athletes perceived them as autocratic and democratic, respectively. Swimmers under coach A’s instructions showed higher cortisol levels, suggesting higher cortisol production related to coaching style. Furthermore, differences in the motivational climate concerning ego (i.e., athletes comparing their ability with that of other athletes) were observed with coach A, whereas differences in motivational climate concerning the task (i.e., athletes comparing their ability with their own past performance) were observed with coach D. Cognitive variables showed negative perceptions affecting athletes’ training experience and performance when they were screamed at or insulted by coach A. There were no gender or age differences in cortisol production or motivational climate. In conclusion, this study suggests that an autocratic coaching style modulates cortisol release in both genders, affecting young elite swimmers’ motivational climate and training experience.

## 1. Introduction

In competitive sports, a coach plays an important role in the “well-being” and performance of athletes. Dominant and autocratic coaching styles could be a source of stress among athletes, affecting mood states and glucocorticoid response [1]. However, some positive effects in the natural environment were also observed, such as uncertainty reduction or intragroup predictability improvement regarding social interactions, especially when the social hierarchy was clearly accepted [2]. Even so, democratic coaching could improve and increase athlete self-confidence, resilience and performance. Coaches should be able to establish contingencies, maintain the intensity of the training process, provide motivation to learn, and offer adequate feedback to achieve better performance [3,4]. Prolonged exposure to aversive stimuli may inhibit the establishment of a balance (e.g., eliciting emotions of frustration, apprehension and anger), affecting athletes’ adaptation process to competition [5].

Some coaches prefer to stimulate democratic and active participation in the training process (e.g., encouraging the cooperation and affective expression within the group to achieve their competitive objectives). Previous authors [6] have suggested that positive dialogue, reflexion and good peer affective social interaction promote better learning, a fact that could be an excellent means by which to reach better technical skills and performance. Previous authors also noted how coaches with different leadership styles could become involved in the training of young athletes, i.e., by stopping training when athletes were facing health problems, modulating hormonal activity or showing a negative affective valence to the training load [7].

Competition outcomes and the perception of rivals as aversive increase cortisol production in athletes [8]. Cortisol activates body metabolism to regulate the athlete’s adaptation to the environmental demands, stimulating gluconeogenesis and mobilising glucose towards skeletal muscle. Cortisol is also an excellent biological marker of psychosocial stress and hypothalamus-pituitary-adrenal (HPA) axis activity, including experiences of social evaluative threat [9]. Negative moods, psychosocial pressure, face-to-face confrontations or defeat frustration have been related to momentary changes in the concentration of circulating cortisol as well [10].

In autocratic leadership and social status seeking, cortisol and testosterone jointly regulate dominance [9]. Coaches with high testosterone and low cortisol levels may be more likely to be dominant, especially when they have previously shown autocratic behaviours [11]. Thus, an autocratic coaching style would become aversive to athletes during training, leading to increased stress response and, over time, making it more difficult for athletes to approach training as an adaptive challenge [12]. Autocratic coaches and learning based on aggressive behaviour, yelling, negative feedback and training overpressure may play negative roles in young athletes’ performance, being linked with discontent and hostility towards subordinates and other components of the group [13]. The aim of the present study was to analyse differences in the psychoneuroendocrine stress responses of high-level young swimmers depending on the implementation of an autocratic or a democratic coaching style. The initial hypothesis was that a democratic coaching style would produce a lower psychoneuroendocrine stress response than an autocratic coaching style.

## 2. Materials and Methods

### 2.1. Participants

Eighteen young, high-level swimmers participated in this study (10 females and 8 males), with the following characteristics: age = 15.33 ± 1.86, and 14.60 ± 2.01 years; BMI = 20.88 ± 1.74, and 21.60 ± 2.88 kg/m^2^; years of experience = 7.50 ± 5.09, and 7.40 ± 3.75, for male and female candidates, respectively. Two male coaches (age = 30 and 26 years, BMI = 25.06 and 31.17 kg/m^2^; with more than 6 years’ experience in high-level competition and performance training programs) also participated in the study. Participants were above 85% in the national ranking; they held previous national titles and were preparing to get a podium in the Spanish Championship some weeks after the time of research.

Coach A was considered autocratic because he usually screamed and insulted the athletes when they did not train hard enough; he just paid attention to swimmers who were outstanding and did not care about the preferences of athletes. In contrast, coach D was considered democratic because he never screamed; he just intervened when the athlete was training poorly, and usually contemplated the preferences of athletes [5,11]. Young swimmers were directly asked how they perceived the training styles of coaches A and D, i.e., as autocratic or democratic (see Section 2.2).

### 2.2. Procedure

First, the procedure was explained to the swimmers’ parents. Informed signed consent was collected according to Helsinki’s Declaration for studies on under-aged participants. Afterwards, separate meetings were conducted to explain the sampling protocol to the participating coaches and swimmers. They were instructed not to drink, eat, or brush their teeth 30 min before sampling. Also, a medical examination to rule out any psychiatric or physical problems, endocrine diseases, use of the contraceptive pill, or consumption of drugs was performed. This procedure was approved by the Ethical Committee of Universidad de Malaga (Spain) with registration number: CEUMA-35-2018-H.

We analysed the psychoneuroendocrine stress response of swimmers in two training sessions: one directed by the autocratic coach and the other directed by the democratic coach. Prior to starting the training sessions, saliva samples were taken from each of the coaches to measure basal testosterone and cortisol levels (i.e., upon waking on a nonworking day) to determine the individual neuroendocrine basal profile of each coach. Saliva samples of swimmers were taken before and after the two training sessions, as well. The first training session was led by the autocratic coach (coach A). After 48 h of recovery without physical activity, the second training session was led by the democratic coach (coach D). Each training session consisted of 6000 m swimming at equivalent intensity levels, divided into three parts: A warm up, consisting of 2400 m swimming at light aerobic intensity; the principal training, consisting of 3000 m high aerobic intensity swimming with instructions and feedback from the coaches; and recovery, consisting of 600 m low aerobic intensity swimming and free swimming style.

Thirty minutes before each training session (before warm-up to reduce the influence of physical activity on neuroendocrine axes) and 30 min after finishing, saliva specimens were collected using Super•SAL™ devices (Oasis Diagnostics^®^ Corporation, Vancouver, WA, USA). Pure•SAL™ devices, with a visual indicator, allowed confirmation that a homogeneous sample of 1–2 mL was obtained. Cortisol concentrations were determined before and after each training session for each swimmer. All participants’ saliva samples were taken between 18:00 and 21:00 h, immediately frozen at −40 °C and stored in the laboratory of the University of Malaga. Samples were centrifuged for 15 min at 3000 rpm and immunoassayed using the Grifols Triturus^®^ (Somagen Diagnostics Inc., Edmonton, AL, Canada) equipment and competitive enzyme immunoassay kits (Diametra, Milan, Italy) with inter-assay coefficients of variation of 10.6% and 8.2%, and 7.4% and 4.6%, sensitivity of 3.28 pg/mL and 0.5 ng/mL, and detection limits of 1000 pg/mL and 100 ng/mL, for testosterone and cortisol, respectively. The samples were immunoassayed twice.

In addition to the collection of saliva samples, psychological variables were measured. The motivational climate perceived factors were determined by the individual sports version questionnaire [14], which analysed the environment generated by coaches A and D. It consisted of 24 items, where 1 applies to “strongly agree” and 5 applies to “strongly disagree” (Cronbach coefficient fluctuated between 71 and 78). The perceived leadership questionnaire [15] about specific coaching styles (i.e., two factors: autocratic or democratic behaviour) with 14 items, where 1 meant “always” and 5 meant “never” (Cronbach coefficient was 79−93), was also applied.

The hormonal baseline (awakening on a resting day) concentrations of each coach to analyse their basal testosterone concentration values indicated that coach A showed higher concentrations of testosterone (150 pg/mL) than coach D (80 pg/mL). Coach A’s concentration was considered high (close to 95%, characterised in serum [16]), whereas it was medium for coach D (close to 50%). Serum testosterone and salivary testosterone showed a very high correlation coefficient (i.e., higher than 92) in prior studies [17]. Cortisol concentrations were considered in a low range, i.e., 2.03 and 2.28 ng/mL for coaches A and D, respectively. This hormonal profile fitted with van der Meij et al.’s [11] study about the testosterone-dominance relationship in autocratic leaders.

### 2.3. Data Analyses

All statistical analyses were performed with the statistical package SPSS^®^ 20.0 (IBM Co, Armonk, NY, USA). Normality was tested by the Shapiro-Wilk test showing the non-parametric distribution of hormonal levels. Hormonal levels were log-transformed to fulfil the homogeneity assumption (non-transformed data were depicted in the figure to facilitate comparison with prior studies), following the statistical procedure performed by Aguilar et al. [10]. Repeated measures ANOVA was used to analyse differences in cortisol levels over time, and the effect size was measured using coefficient η^2^_p_. A Wilcoxon rank paired test was performed to determine whether there were differences in athlete’s hormonal response before and after each training session, and the effect size was measured using Cohen’s *d* test. The Mann-Whitney U test was performed to analyse gender or age differences in psychological variables and self-report scales. Post hoc power effect was also performed to detect 1-β error probability. The level of significance was set at *p* < 0.05.

## 3. Results

**Figure 1:**
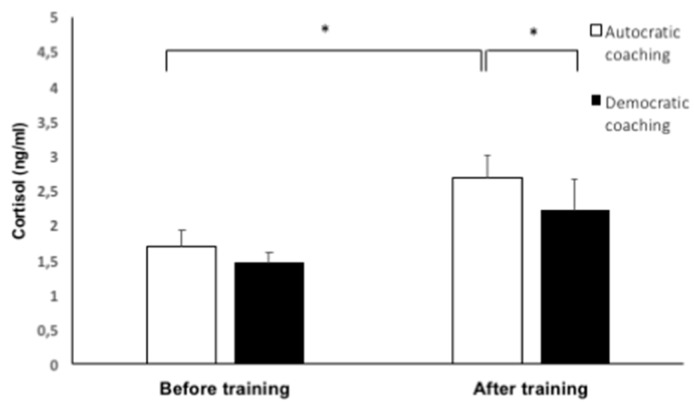
Mean ± SE salivary cortisol depending on coaching style. * *p* < 0.05.

Repeated measures ANOVA was performed to analyse swimmers’ hormonal level differences over time. Before training, the cortisol levels in both training sessions were similar; however, we found significant differences in cortisol production and a high effect size when swimmers were trained by coach A (F_(1,16)_ = 3298, *p* < 0.03, η^2^_p_ = 0.18). A Wilcoxon ranked paired test was also performed to analyse differences in cortisol concentrations after training. Salivary cortisol concentrations were higher after training with coach A (Z = −2.17, *p* < 0.03, Cohen’s *d* = 0.57), compared with coach D. Differences between before and after training were also observed in cortisol under coach A’s training (Z = −1.97, *p* < 0.05, Cohen’s *d* = 0.86), showing a higher stress response. The results showed that swimmers under coach A’s instructions had greater hormonal activity compared to under coach D (Figure 1). Short sample sizes usually were related to type-II error, but this study showed differences in cortisol and motivational climate with power effects (1-β error probability) over 0.90. The Mann-Whitney U test showed no differences by gender or age in hormonal response and self-determination.

Table 1 shows motivational climate and coaching style perceived in training days. Under coach A’s instructions, athletes increased their motivation towards ego (Z = −3.26, *p* < 0.001, Cohen’s *d* = 1.69). However, under coach D’s instructions, swimmers increased their motivation towards the task (Z = −3.42, *p* < 0.009, Cohen’s *d* = 0.64).

Swimmers did not consider coach A to be a leader, and his coaching style was perceived as autocratic (Z = −2.58, *p* < 0.01, Cohen’s *d* = 1.69). In contrast, swimmers perceived coach D as democratic (Z = −2.14, *p* < 0.009, Cohen’s *d* = 0.68). Self-reports on athletes’ experiences when they were trained by both coaches showed higher satisfaction when they were trained by coach D (Z = −2.07, *p* < 0.038, Cohen’s *d* = 0.94), and they felt embarrassed when coach A shouted and insulted them while giving feedback in the training session (Z = −2.16, *p* < 0.031, Cohen’s *d* = 0.63).

## 4. Discussion

The aim of the study was to analyse differences in the psychoneuroendocrine response patterns of high-level young swimmers upon exposure to autocratic and democratic coaching styles. The initial hypothesis was fulfilled, since the democratic coaching style produced a lower psychoneuroendocrine stress response than the autocratic coaching style.

When participants were under pressure (e.g., following coach A’s negative feedback), cortisol production increased according to the self-determination theory [18]. The perceived behaviour of their coach affected the athletes’ coping styles and well-being; therefore, aversive consequences could be associated with negative autocratic feedback as well. It is important to note that perceiving this activity as unpleasant has physiological, psychological and affective consequences on athlete performance and health [19]. In this line, previous studies have suggested that coach behaviour may influence athlete behaviour, increasing exhaustion, affecting self-confidence, and even resulting in the acceptance of cheating and deception to get their individual objectives [20]. One study with novice dancers suggested that an autocratic style could boost intrinsic motivation [21]. However, in this study, the aggressive, autocratic style was considered least satisfying and effective by the young swimmer sample [22].Then, there is a need to find a fitting coaching style according to athlete needs.

The perception of being exposed to an autocratic coach showed increased cortisol levels in preactive swimmers before training. This anticipatory response has also been observed prior to competitions and evaluations, where the perception of threat, uncontrollability and uncertainty triggers a defensive response to prepare a person for any possible hazard [23,24,25]; however, further studies with a large sample size are needed to confirm this. Coach A induced an increase in cortisol concentrations and fostered a climate of “self-demotivation”, negatively affecting the training experience of swimmers. These results are consistent with previous studies, where ego-motivation was associated with anxious states and a greater concern for performance [22]. In contrast, coach D was perceived by swimmers as a democratic coach, and his style of teaching fostered a climate of motivation towards the task and positive and personalised feedback. Similar results have been observed by previous studies [26], which pointed out that motivation towards the task at hand was related to the perception of receiving positive feedback and greater social support from the coach. The inclination towards the task, therefore, could depend on the positive perception that the athletes have regarding their own capacity, yielding better adaptive performance than a climate towards ego [27,28].

Training is a long-term process where the acquisition of new technical, physiological and psychological skills depends on the psychophysiological status of the athlete. The periodisation of these physiological and psychological skills in the training load, as well as a correct dosage of technical skill training, would allow the coach to assist in the best progression of the athletes [29,30]. In this line, it is important to highlight the need of a climate of trust and personal security to enhance desire and freedom to learn and communicate without fear of failure. Exchange with the environment causes the reconstruction of cognitive schemes, and failure is an inestimable source in this exchange, because it gives rise to the appearance of cognitive dissonances that cause the reconstruction of cognitive schemes [31]. Skills failure is a good option to learn new strategies and probe different motor skills to find other performance solutions. An environment focused on punishment increases the tendency to hide errors, making it more difficult to acquire new learning based on the search for effective individual solutions. A possible explanation of cortisol increases in the swimmers of the present study was stimuli associated with learning. Some reinforcement contingencies are associated with emotional response; for example, reward extinction or omission is related to frustration [32]. When young athletes train hard, they expect positive feedback and verbal reinforcement from their coaches. Negative feedback and verbal reinforcements could be perceived as punishment, increasing cortisol production due to stress and frustration [10]. In learning contexts, negative feedback or insults did not help to improve feedback-based tasks, especially under psychosocial stress [33].

Swimmers enjoyed training more and perceived a greater personal effort when they were directed by a close and affective coach, which gave rise to a climate of motivation directed towards the task (Coach D). This subjective perception fits previous research works [34], where motivation was related to the task with a greater perception of enjoyment and satisfaction during training. The athletes considered that their performance was linked to a style of democratic teaching rather than an autocratic style. Previous authors [35] have suggested that the self-evaluation of improvement and competitive performance by objectives were more frequently observed when there was a climate of motivation towards the task. This perception of improvement and performance established by the affective-democratic feedback promoted by coach D and the climate towards the task firmly departed from the perception of failure related to each verbal warning received by coach A, which was related to an ego motivation climate, with negative feedback oriented towards punishment [36]; a tendency of coach A was consistently observed to promote a climate of motivation towards ego and, consequently, a probable impairment of individual self-evaluation, which should be confirmed in subsequent studies.

### Limitation of the Study

This study presents some limitations. Firstly, it was very difficult to find high level athletes to be exposure at identical training with equivalent loads in the same days. Results must be considered as new knowledge to be confirmed or refuted in longitudinal research works. Second, hormonal samples were taken between 18:00 and 21:00 h when circadian cycles were decreasing; this must be taken into account to replicate this study in the future. Finally, a testosterone analysis of swimmers would provide us with a better compression of this complex response, as well as a performance analysis of each training session.

## 5. Conclusions

In conclusion, a direct relationship between autocratic coaching style and higher neuroendocrine activity was observed in young, high-level swimmers. It was confirmed by the momentary increase in circulating cortisol concentration before and after training when young swimmers were under coach A’s instructions. Along with the momentary fluctuations of this hormone, there was a greater predominance of an ego-motivational climate, and consequently, a negative influence on related psychological factors (e.g., deterioration of training day experience) or affective factors (e.g., frustration linked to perceived verbal reprobation after failures). An autocratic coaching style could lead to a deterioration in the athlete–coach relationship, and could be unfavourable to the progress of young, promising swimmers over the medium term, affecting their neuroendocrine response patterns, self-confidence and motivational climate.

## Figures and Tables

**Table 1:** Mean ± SD self-informed differences in perceived coaching styles and motivational climate in young swimmers on training days.

Self-Informed Differences inperceived Coaching Styles	Coach A	Coach D	*p*
Autocratic	15.25 ± 3.15	12.13 ± 3.44	0.001
Democratic	26.23 ± 6.94	33.13 ± 6.01	0.001
Motivational ego climate	43.06 ± 9.84	30.25 ± 4.28	0.01
Motivational task climate	39.81 ± 7.59	46.13 ± 5.60	0.009

## References

[1] Oberle E., Schonert-Reichl K.A. (2016). Stress contagion in the classroom? The link between classroom teacher burnout and morning cortisol in elementary school students. Soc. Sci. Med..

[2] Magee J., Galinsky A.D. (2008). Social hierarchy: The self-reinforcing nature of power and status. Acad. Manag. Ann..

[3] Olusoga P., Butt J., Hays K., Maynard I. (2009). Stress in elite sports coaching: Identifying stressors. J. Appl. Sport Psychol..

[4] van Duijvenvoorde A.C., Peters S., Braams B.R., Crone E.A. (2016). What motivates adolescents? Neural responses to rewards and their influence on adolescents’ risk taking, learning, and cognitive control. Neurosci. Biobehav. Rev..

[5] Keatlholetswe L., Malete L. (2019). Coaching Efficacy, Player Perceptions of Coaches’ Leadership Styles, and Team Performance in Premier League Soccer. Res. Q. Exerc. Sport.

[6] Light R.L., Harvey S. (2017). Positive pedagogy for sport coaching. Sport Educ. Soc..

[7] Nazarudin M., Fauzee O., Jamalis M., Geok K., Din A. (2009). Coaching leadership styles and athlete satisfaction among Malaysian University Basketball team. Res. J. Int. Stud..

[8] Jones A.C., Josephs R.A. (2006). Interspecies hormonal interactions between man and the domestic dog (*Canis familiaris*). Horm. Behav..

[9] Viau V. (2002). Functional cross-talk between the hypothalamic-pituitary-gonadal and-adrenal axes. J. Neuroendocrinol..

[10] Aguilar R., Jiménez M., Alvero-Cruz J.R. (2013). Testosterone, cortisol and anxiety in elite field hockey players. Physiol. Behav..

[11] Van der Meij L., Schaveling J., van Vugt M. (2016). Basal testosterone, leadership and dominance: A field study and meta-analysis. Psychoneuroendocrinology.

[12] Ntoumanis N., Thøgersen-Ntoumani C., Quested E., Hancox J. (2017). The effects of training group exercise class instructors to adopt a motivationally adaptive communication style. Scand. J. Med. Sci. Sports.

[13] Lippitt R., White R., Cartwright D., Zander A. (1968). Leader Behavior and Member Reaction in Three Social Climates. Group Dynamics: Research and Theory.

[14] Balaguer I., Guivernau M., Duda J., Crespo M. (1997). Análisis de la validez de constructo y de la validez predictiva del cuestionario de clima motivacional percibido en el deporte (PCMSQ-2) con tenistas españoles de competición. Rev. Psicol. Deporte.

[15] Chelladurai P., Saleh S. (1980). Dimensions of leader behavior in sports: Development of a leadership scale. J. Sport Exerc. Psychol..

[16] Brambilla D.J., Matsumoto A.M., Araujo A.B., McKinlay J.B. (2009). The effect of diurnal variation on clinical measurement of serum testosterone and other sex hormone levels in men. J. Clin. Endocrinol. Metab..

[17] Arregger A.L., Contreras L.N., Tumilasci O.R., Aquilano D.R., Cardoso E.M. (2007). Salivary testosterone: A reliable approach to the diagnosis of male hypogonadism. Clin. Endocrinol..

[18] Bartholomew K.J., Ntoumanis N., ThØ gersen-Ntoumani C. (2009). A review of controlling motivational strategies from a self-determination theory perspective: Implications for sports coaches. Int. Rev. Sport Exerc. Psychol..

[19] Oldehinkel A.J., Ormel J., Bosch N.M., Bouma E.M., Van Roon A.M., Rosmalen J.G., Riese H. (2011). Stressed out? Associations between perceived and physiological stress responses in adolescents: The TRAILS study. Psychophysiology.

[20] Roxas A.S., Ridinger L.L. (2016). Relationships of coaching behaviors to student-athlete well-being. High. Educ. Politics Econ..

[21] Castillo D.B., Espinosa A.A. (2014). Autocratic and participative coaching styles and its effects on students’ dance performance. Int. J. Learn. Teach. Educ. Res..

[22] Pensgaard A., Roberts G. (2002). Elite athletes’ experiences of the motivational climate: The coach matters. Scand. J. Med. Sci. Sports.

[23] Belinchon-deMiguel P., Clemente-Suárez V.J. (2018). Psychophysiological, Body Composition, Biomechanical and Autonomic Modulation Analysis Procedures in an Ultraendurance Mountain Race. J. Med. Syst..

[24] Beltrán-Velasco A.I., Ruisoto-Palomera P., Bellido-Esteban A., García-Mateos M., Clemente-Suárez V.J. (2019). Analysis of psychophysiological stress response in higher education students undergoing clinical practice evaluation. J. Med. Syst..

[25] Sánchez-Conde P., Beltrán-Velasco A.I., Clemente-Suárez V.J. (2019). Influence of psychological profile in autonomic response of nursing students in their first hospital clinical stays. Physiol. Behav..

[26] Smith S.L., Fry M.D., Ethington C.A., Li Y. (2005). The effect of female athletes’ perceptions of their coaches’ behaviors on their perceptions of the motivational climate. J. Appl. Sport Psychol..

[27] Boixados M., Cruz J., Torregrosa M., Valiente L. (2004). Relationships among motivational climate, satisfaction, perceived ability, and fair play attitudes in young soccer players. J. Appl. Sport Psychol..

[28] Jowett S., Lavallee D. (2007). Social Psychology in Sport.

[29] Clemente-Suárez V.J., Ramos-Campo D.J. (2019). Effectiveness of Reverse vs. Traditional Linear Training Periodization in Triathlon. Int. J. Environ. Res. Public Health.

[30] Clemente-Suárez V.J., Fernandes R.J., Pelarigo J.G., Arroyo-Toledo J.J., Vilas-Boas J.P. (2018). Do traditional and reverse swimming training periodizations lead to similar aerobic performance improvements?. J. Sports Med. Phys. Fit..

[31] Pérez A., Soto E., Sola M., Serván M. (2009). Aprender Cómo Aprender. Autonomía y Responsabilidad: El Aprendizaje de los Estudiantes.

[32] Rolls E.T. (2005). Emotion Explained.

[33] Petzold A., Plessow F., Goschke T., Kirschbaum C. (2010). Stress reduces use of negative feedback in a feedback-based learning task. Behav. Neurosci..

[34] Vazou S., Ntoumanis N., Duda J.L. (2005). Peer motivational climate in youth sport: A qualitative inquiry. Psychol. Sport Exerc..

[35] Pensgaard A.M., Duda J.L. (2003). Sydney 2000: The interplay between emotions, coping, and the performance of Olympic-level athletes. Sport Psychol..

[36] Balaguer I., Castillo I., Tomás I. (1996). Análisis de las propiedades psicométricas del Cuestionario de Orientación al Ego ya la Tarea en el Deporte (TEOSQ) en su traducción al. Psicológica.

